# Non-financial barriers in oral health care: a qualitative study of patients receiving opioid maintenance treatment and professionals’ experiences

**DOI:** 10.1186/s13011-021-00379-6

**Published:** 2021-05-17

**Authors:** Siv-Elin Leirvaag Carlsen, Katja Isaksen, Lars Thore Fadnes, Ole Jørgen Scheie Lygren, Anne Nordrehaug Åstrøm

**Affiliations:** 1 Department of Addiction medicine, Haukeland University Hospital, PO Box 1400, 5021 Bergen, Norway; 2 Department of clinical dentistry, University of Bergen, PO Box 7804, 5020 Bergen, Norway; 3 Institute of Global health, University of Bergen, PO Box 7804, 5020 Bergen, Norway; 4 Oral Health Centre of Expertise in Western Norway, PO Box 7900, 5020 Bergen, Norway

**Keywords:** Oral health, Opioid maintenance treatment, OMT patients, Dental health care workers, Barriers

## Abstract

**Background:**

People with substance use disorders often have poor oral health, which can negatively impact their quality of life. Since 2005, patients receiving opioid maintenance treatment (OMT) in Norway have been eligible for free oral health care services offered through public oral health clinics. Despite a large need for oral health services amongst patients in OMT, figures suggest that the use of these services is low amongst this patient group. It has been unclear which barriers that contribute to this. This qualitative study explores the underlying barriers to the use of oral health care services amongst patients in OMT, from the perspective of the patients as well as dental health care workers (DHW).

**Methods:**

Through a combination of focus group interviews and individual interviews, data were collected from 63 participants; 30 patients in OMT and 33 DHW. Thematic analysis identified key themes for the use (or not) of oral health care services amongst patients in OMT.

**Results:**

Both individual and structural barriers prevent OMT patients from using the free oral health care services offered to them. These barriers include struggling to attend appointments, anxiety and fear of dentists, discrepancies between patients’ expectations and the services offered and perceived stigma. OMT patients’ lack of information regarding their rights and access to oral health services was also a barrier, as was DHWs’ lack of knowledge and information of the OMT system and what they can offer patients.

**Conclusions:**

OMT patients face several barriers in accessing and using oral health care services. However, through a number of relatively simple measures, it is possible that the use of oral health services amongst OMT patients can be increased.

## Background

Substance use has many health-related consequences, including hepatitis [1, 2], tuberculosis [3, 4], soft tissue infections [5, 6], impaired quality of life [7], and anxiety and depression [8–10]. Although, oral health problems and oral diseases have been identified as detrimental side effects of substance use, they have received comparatively little attention in the substance use literature [11–14]. Moreover, research on oral health specifically for patients in opioid maintenance treatment (OMT) is inadequate.

Adequately functional dentition is defined as 20 well distributed teeth, the sum of own teeth, a bridge joint and remaining roots over two millimetres [15]. A 33 years follow-up study among males with long-term substance use disorder (SUD) in USA, found the average number of remaining teeth to be 16 [16]. Similarly, a Norwegian pilot study amongst 29 people with SUD, found an average of 17 functional teeth, with 2.7 decayed and 11 missing teeth [17]. In addition, more than half of the participants were dissatisfied with the appearance of their teeth and mouth and almost half of them reported adverse effects of their oral health conditions on their quality of life [17]. Similarly, in a study by A.N. Åstrøm, Virtanen, Özkaya, et al. [18], of 167 Norwegian OMT patients, half presented with 20 or less remaining natural teeth, 78 % were dissatisfied with oral health and 61 % reported at least one oral impact on daily performances.

A study amongst homeless injecting substance users in USA found that although 63 % reported a need for oral health care in the past six months, only 27 % of them had sought care [19]. Studies show that lack of financial resources is an important factor for low prioritisation of oral health among patient with SUD [20]. However, in most counties in Norway, oral health care service are free of charge for patients in OMT and people with SUD. Nevertheless, only 42 % of investigated OMT patients reported having visited a dentist regularly/annually [18].

People with SUD have been found to have poor oral health in comparison with the general population or comparable groups [11, 21, 22]. For example, prostheses are more common in people with SUD than the general population; 34 % vs. 8 % respectively [17]. Specifically, oral problems have frequently been reported amongst people with opioid dependence [23], and is often associated with personal neglect, poverty, poor nutrition, and high consumption of sweetened food [13, 16, 23, 24]. People with SUD report disproportionately high rates of caries, periodontal disease and xerostomia (dry mount) [11, 12, 15, 23, 25]. Furthermore, people recovering from SUD attribute “rotten teeth”, toothache and tooth loss to the methadone used in OMT [12]. In addition, OMT patients demonstrated an increase in consumption of sugary food after four years in treatment [26]. Thus, the literature suggests that in addition to using illicit substances, several prescribed medications and the use of methadone/buprenorphine can lead to oral disease and problems such as tooth decay and dry mouth [12, 25].

There might be individual and/or structural barriers related to OMT patients’ use of oral health care. Individual barriers, such as having low self-esteem, continuous use of substances [12], dental phobia, low pain tolerance, anxiety, and repeated trauma in previous oral treatments [27] are highlighted as barriers for people with SUD to seek oral treatment. Furthermore, structural barriers e.g. poor accessibility of services [12], social background, inadequate finances and lifestyle challenges [11] are also obstacles for seeking oral treatment. In addition, stigmatising attitudes [28], which refers to an extremely discrediting attribute [29] e.g. substance use, can also be a barrier for seeking treatment [30–32].

Despite a high degree of oral health issues and a need for oral health care service [33], people with SUD often neglect their health problems [24, 34]. This carelessness often results in missed opportunities for early diagnosis [34, 35]. However, studies have shown that people with SUD primarily visit dentists when they are in pain and discomfort [12, 15, 27], and limited access to oral health care services are also reflected by the fact that people with SUD have fewer restorations of teeth [11].

It has been unclear which barriers that contributes to limited utilisation of oral health care services despite poor oral health among people with SUD. The current study builds on two quantitative studies conducted amongst patients in OMT and DHWs. These studies sought to map the overall dental health status and experiences of dental health services amongst OMT patients [36], and examine DHWs’ experience and attitudes towards treatments of patients in OMT [35, 37]. The current study aim to examine OMT patients’ and DHWs’ perceptions of barriers related to the use of oral health care services among OMT patients. Considering that cost is often perceived one of the main barriers to (the) use of oral health care services [20], this article explores the reasons for low use of oral health care services in a context where this barrier has been removed; i.e. in Norway.

## Methods

### Setting

The current study was undertaken in eight OMT outpatient units and in four public oral health clinics in Bergen, Norway. Bergen has eight suburbs and about 900 patients recieve OMT in the municipality [38]. Oral health clinics are located in different districts within Bergen and each clinic is responsible for providing services to the population of one or more of the eight suburbs.

In Norway, people with SUD enrolled in OMT programmes have been provided with free oral health care at public oral health care services, by the welfare state and the county councils since 2005 [17, 39]. However, the oral services offered to each patient is assessed against the severity of the disease, the benefit of treatment and whether the benefit is proportionate to the cost. According to the Norwegian Act on oral health care services § 1–3 [39], various groups are placed in order of priority for free oral healt care. The prioritised groups are, respectively, (a) children and people < 18 years old, (b) intellectually disabled people, (c) elderly and individuals with long-term disease or disabled living in institutions or in need of home nursing, (d) adolescents aged 19–20 years and (e) other groups that the county council priorities [39]. People with SUD are categorised in the lowest priority group (e), along with those exposed to torture and abuse, patients with odontophobia, individuals who are incarcerated for over 3 months, refugees and asylum seekers [40]. Therefore, each county council decides whether OMT patients are given priority for free oral health care or not. Bergen lies in the county council of Vestland, which has prioritied free oral health care for OMT patients.

### Study design

Qualitaitve method by semi-structured interviews were used to understand OMT patients’ and DHWs’ perspectives on barriers in oral health care services. Data were collected via focus group interviews and individual interviews of both groups, separately, during the period November 2019 to January 2020. Focus group interviews was conduced first, and allowed the participants to converse the topic in the context of their own and shared experiences. The individual interviews were conducted on the basis of the researchers desire to explore more deeply specific perspectives and clarify issues that had emerged in the focus group interviews. The individual interviews provided some extra in-depth information on some topics. However, a meaning saturation was reached when the narratives from the individual interviews repeated the narratives from the focus group interviews. This was decisive for the number of individual interviews.

 The focus group interviews consisted of six to eight participants, and the focus group interviews and the in-depth interviwes lasted 45–60 min. Participants provided written informed consent based on details of the study, the confidentiality of the provided information and their right to withdraw from the study at any time.

### Sample

 A targeted recriutment of DHW to participate in focus group interviews was made by recruiting dentists, dental secretaries and dental hygienists from the four public oral health clinics in Bergen with the highest proportion of registered OMT patients. The majority of DHW participants were dentist, with few dental hygienists; this was due to the scope of work of dental hygienists (tooth cleaning and polishing) which falls outside the typical OMT patient’s needs. Furthermore, females were over represented amongs the dentists. This is because the gender distribution among dentists in Vestland County Council is approximately 80 % female [35], while nationally there is a more even gender distribution, 52 % female and 48 % men [41].

 OMT participants were recruited to focus group interviews either by research nurses working at OMT outpatient clinics, or by staff at two local SUD care centers. In Norway, the gender distribution in OMT is 70 % men and 30 % women [42], which also is reflected in the OMT sample in this study.

 Participants for individual interviews were recruited through snowball sampling. Participants recruited for DHW focus groups interviews were asked if they had colleagues who could be interested in participating in an individual interview. For individual OMT interviews, research nurses at the OMT clinics were asked if they knew patients who could be interested in participating. In order to avoid duplication and to obtain a broad sample, none of the participants in the focus groups were interviewed individually.

### Participants

In total, 63 individuals participated in this study, 30 OMT patients and 33 DHW. Nine focus group interviews were conducted, with a total of 56 participants. Four focus groups interviews were with DHW (n = 29) and five with OMT patients (n = 27). Individual interviews were conducted with three OMT patients having between five to 15 years of experience from OMT. Four individual interviews were with DHW with 4.5 to 15 years of experience treating OMT patients. The DHW individual interviews were all with dentists.

In the DHW sample, females were overrepresented (28 females, five males) while males were overrepresented in the OMT sample (19 males, 11 females). DHW in the focus group interviews had on average 9.6 years of experience with OMT patients, ranging from two months to 15 years. Among DHW, 16 participants were dentists, 11 were oral secretary, and two were dental hygienists. On average, patients had been in OMT for 7.3 years, ranging from two months to 21 years.

### Data collection

Semi-structured interview guides were developed for DHWs and OMT participants and were adjusted for use in both the focus groups and individual interviews. The interview guides were based on previous research conducted by the research group [18, 35–37] and results from national and international research.

 The OMT interview guide focused on patients’ feelings about their teeth, impact of oral health on daily life, knowledge about the oral services available to them, past experiences of oral treatments while in OMT, expectations of oral health care services and opinions on why few OMT patients make use of free oral healt services. The DHW interview guide focused on barriers and facilitators in providing oral health care for OMT patients and on DHWs experiences and attitudes towards this group of patients. The following questions were included: (1) experiences in treating OMT patients, (2) challenges faced treating them, (3) potential communication issues with this group of patients, (4) knowledge/competence about OMT, and (5) thoughts and experiences of barriers for OMT patients use of free oral healt service.

All interviews took place in a private space. The DHW focus group interviews were held during clinic hours, typically in the clinic’s lunch/break room. Each clinic received financial compensation for the estimated loss of hourly income. The focus groups interviews with patients were arranged in OMT outpatient clinics or in the SUD care center, and lasted between 30 and 45 min. Similarily, OMT participants received compensation for study participation (gift card). Two independent researchers conducted the interviews and data analysis. Seven individual interviews were completed, three with OMT patients and four with DHW.

A study report was submitted to Vestland County Council in January 2020. The Norwegian Centre for Research Data granted ethical approval (NO 59,417).

### Data analysis

All interviews were conducted in Norwegian and audio-recorded. Interviews were transcribed verbatim, translated into English. The English transcripts were reviewed for accuracy by a Norwegian native speaker (who was fluent in English) by listening to the (Norwegian) recording and reading the (English) transcripts.

The researchers opted for a thematic analysis to identify, analyse and report themes [43]. We conducted a “bottom-up” analysis, and by choosing a qualitative approach we aimed to elaborate the findings from quantitative studies conducted amongst patients in OMT and DHWs in 2018 [18, 35–37]. Throughout the data collection process, the researchers continually discussed their thoughts on the recurring themes emerging in the focus group interviews and individual interviews. However, formal thematic codes were not developed until the data collection was completed. This approach was chosen in order to allow for the identification of relevant themes of interest, which may not have been covered in the interview guides. After completing the data collection, both researchers, separately, listened to and read all the transcripts to familiarize themselves with the data, identified dominant themes and developed preliminary codebooks both manually and using NVIVO 12.0 software [44]. The researchers discussed the themes they had each identified and consensus was reached by consolidating similar themes and removing and/or recoding others. This allowed for a final codebook which was applied to the data. NVIVO 12 was used to generate the main categories and subcodes.

## Results

Both DHW and OMT patients pointed out potential barriers to get OMT patients to the clinic. Several reasons for not attending appointments were given. When asked why they did not go to the dentist, an OMT participant answered:

*Lack of bus tickets, lack of money to get to the dentist, sudden need for drugs instead of the dentist.* (OMT focus group, male)

Both OMT patients and DHW highlighted that lack of structure in everyday life and time management problems were a reason why patients did not make it to appointments. Similarly, contacting the patients was often cited as being difficult as they often lose their phones, or often changing phone numbers without updating contacts such as the oral health clinic. Some patients have no permanent address and thereby lack the opportunity to receive appointments by post. In addition to these logistical issues, DHWs confirmed that missed appointments, no-shows and long periods of treatment absence were common among OMT patients. This also had direct consequences on the effects of the treatment given, as expressed by a DHW:

*(…), but what can be very challenging with these ones [OMT patients] is that they don’t show up for appointments right. They make an appointment and then they get so scared that they don’t come and we don’t manage to help them because we don’t get them here. That can be a big problem. We have offered nitrous oxide treatments [used for its anaesthetic and pain reducing effects, commonly called laughing gas] and they don’t come to those appointments and we try and try, so we use a lot of resources…* (DHW focus group, female).

This perspective was extended by another DHW respondent;

*…it can go in waves. Sometimes they are in* ***very*** *good condition and then [in other periods] they can be completely gone. Then they tear down the things [treatment] the dentist has just built up.* (DHW focus group, female)

The quotes illustrates that OMT patients can be unstable in the course of oral treatment, and treatment is not always completed. Consequently, OMT patients may experience a lower priority at DHWs, as DHWs prioritise other patient groups.

With the abovementioned knowledge as a backdrop, barriers to access and use of oral health care services were linked to the following themes: (1) anxiety and dental fear/phobia amongst patients, (2) expectations versus available services, (3) knowledge deficits among DHWs and OMT patients, and (4) perceptions of shame and stigma.

### Anxiety and dental fear/phobia amongst patients

The impact of fear and subsequent no-shows was acknowledged by OMT patients, and one patient conveyed:

*(…) it took quite a long time before I was finished [with the oral treatment], but that was a little bit my fault as well, because I didn’t show up to all the appointments, because I am scared.* (OMT focus group, female)

This perspective was extended by another OMT patient:

*… dental phobia means a lot, like if I just see that injection [the needle], you can forget about it, I’ll then just leave, I’m not having that in my mouth.* (OMT focus group, male)

Anxiety and dental fear/phobia were reasons not only for missing scheduled appointments, but also for not making new ones or constantly rescheduling appointments. According to DHWs, OMT patients had more anxiety compared to other groups of patients. Due to painful treatments in the past, often from school dentists, the anxiety was particularly high among the oldest OMT patients; likely due to the dated treatment approach and equipment used by dentists at that time. The reasons mentioned by patients included fear of injections, low pain threshold and fear of losing teeth. DHWs also confirmed this:

*… maybe they have bad experiences from before. Maybe they experienced from when they were young, like the anaesthetic injection was painful. I feel that a lot of them talk about experiences from their childhood when they had bad oral health and there has been a lot of treatments, for example extractions and urgent/acute treatments, it’s these kinds of treatments that are often scary and painful for a child. I feel like there are many who tell me that they have many of those kinds of experiences from when they were children as well.* (DHW, individual interview)

However, some patients received help to enable them to complete treatment that may otherwise not have been possible. DHWs described using extra time to reassure the patient by explaining the procedure and using extra anaesthetics before treatment. Anaesthetic and pain reducing treatments such as nitrous oxide were used in extreme cases. However, it was interesting to note that these additional efforts were not offered by all dentists, despite patients reporting a need for them. For example, nitrous oxide treatment was not available at all of the oral health clinics. As expressed by an OMT patient:

*Well, now I’ve found a dentist who is ok and gave me nitrous oxide so I managed to attend and lie there [in the dentist chair]. Otherwise, I wouldn’t even manage to go to a dentist’s office. I got such fear and such panic…* (OMT focus group, female).

The DHWs is aware of the issue of anxiety, and some have implemented measures to address this, which may be helpful for some patients. However, anxiety can be a major barrier for OMT patients’ oral treatment, from making appointment, attending, and completing treatment.

### Expectations versus available services

OMT patients often reported that they only went to the dentist when they had an acute problem and expected the dentist to fix the “problem” rapidly, in one or few visits. Despite this expectation, the patients often explained that the time between each appointment was too long. Hence, there was a mismatch between patients’ extensive treatment needs that required long-term treatment and, according to DHWs, patients’ unrealistic expectations to the time needed for complex treatments, as one DHW conveyed:

*Yes, it is a bit like you are saying they [OMT patients] often have expectations like “yeah can’t you [the dentist] just finish” or “can’t you do as much as possible today”. I mean they have the wrong expectations about how long this will take…*.

(DHW focus group, female)

In addition to the OMT patients’ impatience with oral treatment, it seemed that patients often receive a lack of information about why treatments are time consuming, as expressed by an OMT patient:

*Well it’s way too much time between each appointment when on OMT. It’s crazy. I don’t understand why I have to wait like 1.5 months before the next appointment.* (OMT focus group, female)

Another patient extended on this:

*…but I have one thing I am wondering about: when you are in my age, the way I see it, I have to go to the dentist more or less every month until I‘m 6 feet under [and dead]. Is that the way it should be?* (OMT focus group, male)

The data also suggested that patients most often expected replacements of their missing teeth with implants. However, as explained by the DHWs, the provision of permanent prosthetics was not in line with their treatment guidelines and they suggested dentures as a feasible solution. However, there are some occasions where OMT patients have received implants, and this was perceived as unfair and incomprehensible for many of the patients. DHWs also noted that this was challenging to explain to patients. As reported from OMT patients:

*I know many who have had their teeth pinched, and I think that is incredibly unfair, because it’s much better to have dental implants. In the long run it will be cheaper*. (OMT focus group, male)

These aspects were regarded challenging also for DHWs, as one of them stated:

*Yes, we notice that they very often are dissatisfied with the treatment that we can offer them. They want to have for example fixed/permanent “solutions” and we can offer prosthetics for example. Because they are in a very unstable life situation, so we cannot offer them anything other than a prosthetic or denture solution to replace teeth … and that can be pretty challenging to get them to understand. But, these are our guidelines, this is what we can offer you*. (DHW focus group, female)

The expectations OMT patients had did seldom matched the offer from DHW both according to the time aspect and what they were offered e.g. denture vs. implant. These expectations were often based on patients’ lack of knowledge about DHW guidelines or DHWs’ lack of basic knowledge of patients’ life situation and needs.

### Knowledge deficits among DHWs and OMT patients

The DHWs participating in this study treated different groups of patient, and their level of knowledge about OMT patients varied. DHWs reported that it was not common to receive formal training about the OMT system or how to treat OMT patients and a DHW conveyed:

*I can’t think that I had any….teaching or any information. (…) We don’t have any specific competency for OMT patients, but otherwise we do know how to handle different types of medicinal use and how that reacts with what we are doing.* (DHW focus group, male)

As this quote highlight, DHW had little or no training on treatment of OMT patients during or after their education/training, which means that they have a less favourable starting point for dealing with this group of patients. This lack of education or information may influence how DHW meet OMT patients and understand their need, and when asked about their first encounter with an OMT patient DHWs often responded with comments such as:

*Then I though “What is OMT?” hadn’t heard the word before [laughter] didn’t have anything about it in my studies, or otherwise I slept during this tutorial*. (DHW focus group, female)

Furthermore, some DHWs highlighted the need for additional knowledge about the effect of OMT medicine on teeth and/or the degree of appearing intoxicated after ingesting OMT medication. DHWs also described a wish for more information on the commonly used substances and their possible effects on the patient, so that they would know how and with what they could treat them. However, if provided at all, this information was directed mainly at senior dentists or those in managerial positions, resulting in a variation of knowledge between the clinics and its staff. Knowledge about, and communication with, the OMT system was requested by DHWs as this could contribute to improved treatment and communication with patients. One DHW said:

*We know too little, it’s that simple. There’s the whole system, right. And it’s maybe there that we feel that, maybe that could help us with patients in some way. Because the more information we get the easier it is for us to treat people. That’s for everyone, whether it’s psychiatry, the more you know the easier it is, you understand/empathise more with things.* (DHW focus group, female)

This lack of knowledge has direct consequences for OMT patients and their dental treatment as expressed by a patient:

*… once I had not taken a full dose, I had only taken 16 mg [buprenorphine] before I came there [at the oral health clinic]. Well, then she [dentist] couldn’t give me any anaesthetic because she didn’t know how much she could give me, so I had to just come back again…* (OMT focus group, female).

Similarly, the information provided to OMT patients about the oral health treatments available to them, provided by social services and OMT clinics, often seemed inadequate and unevenly provided. For example, patients received limited information, mainly about the right to free oral health care, little or no information about available dentists, how to book an appointment, types of anaesthetic, or the guidelines dentists adhere to. One OMT patients said:

*(…) not everyone is aware that it is free. There are many I know who say: “I cannot afford to go to the dentist”…* (OMT focus group, male).

Furthermore, the lack of clear information allowed for rumors in the substance use community; several OMT patients referred to ‘someone knowing someone’ who got implants and were told they could get it, even if this was not the case. In addition, patients were rarely informed about the possible negative effects of OMT treatment on their oral health, nor how they could prevent these effects. Patients requested such information and guidance regarding oral health care services, as expressed by one OMT patient:

*I haven’t been very good at asking a dentist for example. Well, they don’t usually have any answers either about OMT medications or whatever. I have asked what OMT medicines do to teeth and then she [dentist] didn’t know, or she didn’t have an answer, so that would be interesting to know.* (OMT individual interview, female)

The type of treatment offered to OMT patients seemed to vary, depending on the clinic the patient attended, the degree of knowledge DHW had about OMT patients and how insistent the patients were about a specific treatment, e.g. pain and/or anxiety relief.

### Perception of shame and stigma

 OMT patients reported experiences of prejudice and exclusions based on their oral health status. Being stigmatised had direct impacts on their self-esteem, which also led to feelings of shame and self-stigma.

*I think there is a lot of stigma in relation to, at least at the dentist I now go to, it is very like “oh yes, you’ve been anesthetised twice, yes you did not manage this then”. It’s so arrogant, it’s stigmatisation I feel.* (OMT focus group, female)

Another OMT patient conveyed:

*(…) and then you don’t dare to go to the dentist because you’re afraid of being kind of yelled at, right: “Oh yes, you must not get intoxicated”, and “you’re just a junkie”, so you walk around with such thoughts.* (OMT focus group, male)

These quotes illustrate that OMT patients experienced stigmatising attitudes from DHW. Moreover, patients regarded being offered dentures, rather than implants, as an expression of the dentists’ ill-treatment and stigmatisation. Dentures, which several patients had, were considered embarrassing and largely associated with “old people”. Several OMT patients had a common experience of only being offered dentures because they were OMT patients, and according to one patient, they were offered dentures:

*Because they’ve [dentist] been told to do it as cheap as possible, I asked.*

(OMT focus group, female)

OMT patients also felt that they were being discriminated against and treated like second-class citizens when it came to oral health service provision; many believed other patient groups were offered better quality and more expensive solutions. Patients stated:

...* “you are not exactly met in the same way as you do when you have things [oral health] in order”.* (OMT focus group, female)

Patients explained that they experienced stigma when dentists made prioritisations, treatment plans and decisions without taking into account their wishes or needs, e.g. situations whereby dentists extracted teeth instead of fixing them or without consulting the patient, or where they had to wait extra-long for appointments during follow-up treatment. One OMT patient conveyed:

*One feels very unworthy when they just pull our teeth, I mean, I had a bit of teeth tartar and now it’s starting to turn into cavities, like on my front teeth that makes one feel pretty unworthy. And then, when they just pull them, I mean I get implants, I mean it’s going to be dentures at the end if they don’t sort it out.* (OMT focus group, female)

The communication between OMT patients and DHW could also be a barrier in itself. Satisfactory communication was described as dentists using x-rays slides of patients’ teeth, explaining the procedures, talking comprehensibly and being skilful with the anaesthetic. It seemed that experienced DHWs had developed effective ways to communicate with OMT patients, had an understanding of their situation and were more empathic. However, the majority of OMT patients experienced communication issues such as, dentists being slightly arrogant, commenting patients’ oral status e.g., “*we haven’t seen it this bad before”*, only treating one dental problem at the time, not giving information about impending oral issues, being rude or harsh, and not talking or giving answer to patients’ questions. In this context, one OMT patient conveyed:

*(… ) the dentist doesn’t speak to me, and when I speak to the dentist I don’t always get answers. He ignores me in a way and [only] does the most important, it doesn’t seem like he is out to repair anything. I went for half a year without teeth in the bottom jaw… well hello!?* (OMT focus group, female)

These quotes illustrate the importance of DHWs’ way of communicate. Moreover, in the interviews with DHWs, quotes emerged that illustrated that some DHW had stereotypical attitudes towards the OMT population. One DHW said:

*Like how much can you trust the OMT patients when he comes? (…) You want to believe him and you want to listen to him and believe what he is saying, but we have to be realistic also, can we trust this person?* (DHW focus group, female)

Another DHW expressed:

*(…) they don’t gather relevant information like other people who are let’s say a bit more “functioning”, like reading the paper, listen to the radio and so get a different picture of the situation (…).* (DHW focus group, female)

At the same time, another DHW conveyed:

*(…) I mean we don’t treat them [OMT patients] any differently than the others. I mean we don’t see it like that, but they may* ***perceive*** *that the others get so much more [better treatment] than they do…* (DHW focus group, female).

The DHWs are concerned about not stigmatise OMT patients, however in the encounter with them, OMT patients experience various types of, and in several ways, stigmas that acts as a barrier to benefit oral health care or to continue oral treatment.

## Discussion

The current study found several barriers to why Norwegian OMT patients do not use or benefit from the free oral health care offered to them, which is in extension with previous research [35]. The current study found both individual barriers, e.g. anxiety/dental fear and OMT patients’ expectations, and structural barriers, e.g. stigma, and disparate information, lack of knowledge, and variations in treatment offers.

The data showed that one of the major barriers to treatment for OMT patients was the crucial first step of attending the clinic, start and complete treatment. Numerous OMT patients live complicated and chaotic lives, including active use of legal/illegal substances, making it difficult to comply with appointments at the dental health clinics. Some oral health clinics have developed and implemented their own methods to address this issue, e.g. SMS notification, contact with relatives, social services or contact persons in other services. The benefit of SMS notification is the reminder itself, and that it contributes to OMT patients attending the oral health clinics. The disadvantage is that mobile phone is an insecure communication tool for this group of patients due to frequently changing phone numbers. According to the current study, patients accompanied, by family members or others, to the dentist for oral treatment reported to have less treatment dropout compared to those without such support. A study among 141 DHW and their experiences of oral health care for people with SUD in Norway recently confirmed this association [45]. Accompanying patients to oral treatment might be an approach several clinics should encourage, especially since it seems to have a positive effect on attendance and raises the quality of treatment [45].

In fear of having to pay for the treatment, several did not use the free oral care offer. Even if oral treatment is free of charge for OMT patients in Norway, their lack of economic resources can still be a barrier for use of oral health care services. Our result showed that several patients expected implants, but was offered denture, and in this context, finances can be an obstacle for use of oral health services: the public sector does seldom cover the treatment they want, i.e. implant, and they do not have the funds to cover it themselves. Therefore, they refrain from using the oral health care service, or for those who have financial private support they travel abroad to have their teeth fixed.

In addition to life circumstances, anxiety disorders often co-occur with SUD, which can lead to greater functional impairments [46]. In general, anxiety and odontophobia/dental phobia are found to impact whether patients attended oral health care services or not. Some OMT patients also highlighted that dental fear was linked to needle fear, which is supported by other studies [12, 47]. A Norwegian study among 40 OMT patients found that 46 % reported dental phobia as the reason for not attending oral health care during the last five years [36], illustrating the difficulty in getting patients to the oral clinic. Considering that OMT patients attend outpatient units daily for medicine delivery, despite their high anxiety levels, it is possible that by placing DHWs in OMT outpatient units and low-threshold locations, it could be possible to increase access to preventive and basic oral health care services.

Furthermore, rumours and stories in the substance using community affected OMT patients’ perception and expectations of oral health care services. Some DHWs did not know what OMT entailed or how the OMT medicines effect on oral health. Lack of information often led to frustration in the dentist-patient relationship, and both patients and DHW requested more information.

Lack of knowledge often leads to flawed beliefs and reinforcement of stereotypes. Staff’s education level, years of professional experiences and knowledge about OMT are significantly associated with stigma describing OMT patients [48]. Furthermore, studies have found a relationship between a person’s view of people with disorders and their social distance and response to them [49, 50]. A study among DHW found that their belief that OMT patients cannot complete oral treatment was associated with their negative attitudes [35]. These negative attitudes can lead to reduced collaboration and an evasive approach from DHW towards OMT patients, as well as less successful treatment outcomes [35]. In contrast, having contact and personal experience with people with SUD can lead to reduction of stigmatisation [51]. This is in line with the findings in the current study; experienced DHW with several years of providing treatment to OMT patients experienced less frustration, developed a better communication approach to patients, and experienced less treatment dropout. In addition, they had more knowledge of OMT patients’ needs and had empathy for them. This contradicts findings from a new Norwegian study showing that, compared to experienced DHWs (> 10 years of experience), those with less clinical practice were less likely to experience missed appointments among patients with SUD [45].

In general, the alliance between therapist and patient is essential for successful treatment [52, 53]. Flückiger, Del Re, Horvath, et al. [54] suggest that reduced alliances amongst people with SUD may be explained by damage to the neuro-biological systems required for ideal relational capacity, therefore hindering the patient’s utility of the therapeutic relationship [55]. Conversely, engagement, retention, and reduced distress have been associated to a stronger therapeutic alliance among people in SUD treatment [52]. A study amongst veterans with bipolar disorder, which similar to OMT patients can be seen as a stigmatised group, showed that maintaining a beneficial therapeutic alliance was an important strategy to improve treatment retention and adherence [56]. In addition, individuals feeling respected as a treatment partner and having confidence in their health care environment fared better compared to patients who lacked a voice in their own treatment [56]. In the current study, OMT patients felt they had little or no influence on their treatments and were not perceived as a treatment partner. In addition, they had reduced confidence in oral health care due to experienced stigma. These results highlight a lack of alliance between OMT patients and DHWs. In this context, a lack of responsiveness from the DHWs’ perspective may be due to their high work pressure. A Danish study found that some of the most intense stressors for dentists’ were perceived to be heavy work load, patients being late for appointments and anxious patients [57]. The exception in the current study was the DHW who, through years of experience with OMT patients, had acquired knowledge of how to communicate, treat and inform OMT patients and thereby established efficient alliances with the patient.

Stigma was another important barrier. Fear of negative reactions and being stigmatised are found to be barriers for seeking treatment [32, 58], and opioid dependent individuals are often more severely stigmatised compared to individuals using other illegal substances [30] or suffer from other disorders [59, 60]. Previous studies suggest that experiences of stigmatisation from staff at social and health care facilities is common for OMT patients [51, 61–63]. Furthermore, this stigma often results in self-stigmatisation [64, 65]. The current study showed that OMT patients experienced being obvious or subtle stigmatisation in different contexts by DHW, which is consistent with findings from other studies. A study among patients with a history of addictions and mental illness found that patients were negatively stereotyped, excluded from decision-making process and DHWs avoided or minimised their interactions with them [66]. A small Norwegian study among OMT patients found that 46 % reported that negative experience with how they were met at the last oral treatment was the reason for not attending oral health care service in the last 5 years [36]. These findings adhere with opinions expressed by DHWs. A study examining 163 Norwegian DHWs’ experiences with and attitudes towards treatment of OMT patients, showed that they had slightly negative attitudes toward treatment of OMT patients [35] and 94 % found it difficult, challenging and demanding to treat them due to missed appointments and communication problems [37].

 Narratives from OMT patients indicate that oral health care is of great importance to them. However, implementing oral health programs for patients with SUD can be challenging due to patients’ low priority of oral health [23]. Furthermore, they can be difficult to treat due to lack of compliance with treatment and instructions from oral health care [67]. The current results support these assumptions. Nevertheless, based on the way OMT is organised in Bergen, patients attending outpatient units on a daily basis, it could be particularly suitable to provide information about the right to free oral health care and enable them to utilise this offer. However, this requires that clinicians in OMT address oral health as a topic and have the knowledge needed to provide correct information about patients’ rights to oral health care. It also requires stronger communication channels between the OMT and oral health services systems.

### Study limitation

In Norway OMT is integrated in the specialist health care service and mainly organised as outpatient units. In addition, the OMT model in Norway is varied in that it also allows patients to have medicine dispensed at pharmacies or from home nursing [68]. This may raise question about how applicable our results are in a national and international setting. Despite the fact that OMT models vary and the study was completed in one city, the findings are of general interest as they expand our understanding of the barriers that exist and highlight measures that can easily be implemented to increase the proportion of patients who benefit from free oral health care.

The oral health clinics with the greatest number of OMT patients were selected for this study. OMT patients were recruited from different outpatient units and low-threshold centres in Bergen through voluntary participation. Thus, there is a chance that the participants represent only those who have strong opinions about oral health care services. However, our analyses show that we have gained a range in experiences of barriers related to oral health from both DHW and OMT patients.

## Conclusions

Barriers for OMT patients to benefit from oral health care are many but not insurmountable. Many DHWs requested more specific training on oral health care related to patients with SUD, both in the education system and in additional courses for DHWs. Improving communication and trying to reduce discrepancies in expectations between patients and DHW through training of DHW and other health care workers as well as using appropriate information material available for patients could also be a potential strategy. Furthermore, clinicians in OMT ought to be educated in patients’ rights and limitations to oral health care services so that they can pass this on to patients. There is also a need to use models that reduce the amount of missed appointments while at the same time ensuring that treatments do not extend unnecessarily long. Through increased knowledge and gaining mutual understanding, it is possible to increase use of oral health care services amongst OMT patients.

## Data Availability

The data gathered during the current study are not publicly available due to protections of confidentiality.

## Abbreviations

SUD: Substance use disorder
OMT: Opioid maintenance treatment
DHW: Dental health worker
